# A study of machine learning models for rapid intraoperative diagnosis of thyroid nodules for clinical practice in China

**DOI:** 10.1002/cam4.6854

**Published:** 2024-01-08

**Authors:** Yan Ma, Xiuming Zhang, Zhongliang Yi, Liya Ding, Bojun Cai, Zhinong Jiang, Wangwang Liu, Hong Zou, Xiaomei Wang, Guoxiang Fu

**Affiliations:** ^1^ Department of Pathology, Sir Run Run Shaw Hospital Zhejiang University School of Medicine Hangzhou Zhejiang China; ^2^ Department of Pathology The First Affiliated Hospital, School of Medicine, Zhejiang University Hangzhou Zhejiang China; ^3^ Department of Pathology Hang Zhou Dian Medical Laboratory Hangzhou Zhejiang P. R. China; ^4^ Hangzhou PathoAI Technology Co., Ltd Hangzhou Zhejiang China; ^5^ Department of Pathology The Second Affiliated Hospital of Zhejiang University School of Medicine Hangzhou Zhejiang China

**Keywords:** artificial intelligence, frozen section, histopathology, machine learning, thyroid

## Abstract

**Background:**

In China, rapid intraoperative diagnosis of frozen sections of thyroid nodules is used to guide surgery. However, the lack of subspecialty pathologists and delayed diagnoses are challenges in clinical treatment. This study aimed to develop novel diagnostic approaches to increase diagnostic effectiveness.

**Methods:**

Artificial intelligence and machine learning techniques were used to automatically diagnose histopathological slides. AI‐based models were trained with annotations and selected as efficientnetV2‐b0 from multi‐set experiments.

**Results:**

On 191 test slides, the proposed method predicted benign and malignant categories with a sensitivity of 72.65%, specificity of 100.0%, and AUC of 86.32%. For the subtype diagnosis, the best AUC was 99.46% for medullary thyroid cancer with an average of 237.6 s per slide.

**Conclusions:**

Within our testing dataset, the proposed method accurately diagnosed the thyroid nodules during surgery.

## 1 INTRODUCTION

Over the past few decades, the prevalence of thyroid cancer has increased owing to the improved detection and diagnosis of tiny indolent thyroid tumors.^1^ Fine‐needle aspiration is the primary tool for risk stratification of nodules and guiding treatment. Based on the Bethesda protocol for thyroid cytology reports (from the original 2009 version and revised in 2017), the fine‐needle aspiration cytology (FNAC) divides the thyroid nodules into six categories according to the expected probability of malignancy.^2^ The management of nodules by the FNAC and Bethesda classification system has increased diagnostic accuracy. Hence, papillary thyroid carcinoma is commonly identified preoperatively. However, surgery is still the only definite way to differentiate between malignancy and benign nodules, as cytology cannot reliably separate the indeterminate thyroid nodules.^3^ Frozen sections are widely used to diagnose thyroid cancer during surgery. Thyroid surgeons can choose an individual surgical strategy (restricted primary resection or completion surgery) based on accurate intraoperative pathological diagnosis (IOPD).^4^

One topic that cannot be avoided in this regard is the IOPD of follicular lesions (follicular thyroid adenomas and carcinomas). In most cases, the histological morphology of follicular lesions is essentially the same, and it is generally believed that the two can only be distinguished by identifying the presence or absence of a capsule and/or vascular invasion or metastasis.^5^ FNAC cannot reliably identify follicular thyroid carcinoma based on cell morphology,^6^ and the final diagnosis can only be made by examining the surgical specimen. Peng et al.^7^ reviewed the literature comparing FNAC and frozen sections to evaluate thyroid nodules. Frozen sections appear to have higher specificity and positive predictive value (PPV) than FNAC. Although they proposed not using frozen sections as a diagnostic tool for follicular neoplasms, many additional studies were published later in which the use of frozen sections was evaluated in the same scenario.^8^, ^9^, ^10^ In 2019, Grisales and Sanabria^11^ updated this meta‐analysis and observed a low sensitivity of 43% and a specificity of 100%.

Only experienced pathologists can make accurate pathological diagnoses when classifying and typing thyroid diseases during surgery. However, fewer than 20,000 pathologists are licensed in China. According to the allocation requirements of the National Health Commission for 1–2 pathologists per 100 beds, the shortage of pathologists exceeds 90,000, which is far from meeting clinical needs. Therefore, an alternative tool with quick and precise IOPD is highly desirable.

The rapid advancement of computer hardware and processing algorithms, coupled with the emergence of breakthroughs in artificial intelligence (AI) theory, particularly in deep learning (DL), has significantly impacted various domains of clinical medicine, including medical imaging. Deep learning models, such as convolutional neural networks (CNNs), have demonstrated remarkable performance in tasks such as image classification, segmentation, and detection, which are crucial for medical imaging analysis. With the ability to automatically learn critical features relevant to the target task in the image, deep learning methods have significantly improved the accuracy and efficiency of medical image analysis, making them the mainstream approach in the field of computer image processing. For example, in 2020, Wang et al.^12^ applied AI to eye fundus image reading for preliminary screening, which can significantly improve the current screening efficiency for diabetic retinopathy. In addition to diabetic retinopathy, other ocular conditions can also be diagnosed, including glaucoma, age‐related macular degeneration, cataract, and macular holes.^13^ In 2020, Liang et al.^14^ used a deep convolution neural network to identify the radiological characteristics of the CT images of pulmonary nodules and predict their pathological subtypes. The area under the receiver operating characteristic (ROC) curve (AUC) for patients with squamous cell carcinoma (LUSC) was 0.8792. This method can predict image characteristics, malignant tumors, and pathological subtypes based on non‐invasive CT images and has great application potential in routine clinical workflow. In 2020, Kosaraju et al.^15^ proposed the HipoMap framework, which uses deep learning technology and pathological images of patient tissues for survival analysis and prediction. It is verified using The Cancer Genome Atlas (TCGA) lung cancer data. The AUC of lung cancer classification is 0.96 ± 0.026, the c‐index is 0.787 ± 0.013, and the decision coefficient of survival prediction is 0.978 ± 0.013. This framework is significantly improved compared with the most advanced technologies.^16^

Although there are many applications of AI in medical imaging, research on thyroid histopathology is limited. In 2020, Li et al.^17^ proposed an automatic diagnostic method for thyroid nodules based on a deep learning rule system that utilized a CNN to predict thyroid pathological image patches. This method automatically segments tissue regions, eliminating the significant amount of computation caused by redundant backgrounds, and establishes rules to determine the number of malignant, benign, and uncertain patches to form the final classification. On 259 test slides, the accuracy rates for benign nodules and malignant nodes were 95.3% and 96.7%, respectively, and 42 slides were classified as uncertain. However, this study did not include all subtypes of thyroid disease.

To solve these problems, this study proposes a diagnostic system based on AI deep learning and machine learning technology to identify all subtypes of thyroid diseases during IOPD. Papillary thyroid cancer (PTC), follicular thyroid cancer (FTC), medullary thyroid cancer (MTC), poorly differentiated thyroid cancer (P‐DTC), anaplastic thyroid cancer (ATC), nodular goiter, thyroid adenoma (TA), and thyroid nodules (TN) are included in the system. Slide‐level diagnostic results were provided along with the position of the lesions on the image.

## 2 METHODS

In this study, computer vision technology was coupled with deep learning neural networks and machine learning support vector machines (SVM)^18^ to autonomously recognize the entire disease species of frozen thyroid pathological sections. The frozen portion typically measures 60000 × 60000 pixel and contains a large background area. Blank backgrounds of the slides were removed using a threshold segmentation method for computer vision to improve the image processing efficiency. Then, using an AI algorithm, the useful parts of the slide are learned and understood. The three major phases of the AI algorithm are slide‐level complete disease classification algorithm, patch‐level benign and malignant classification, and patch‐level subtype classification.

### 2.1 Data process

**FIGURE 1:**
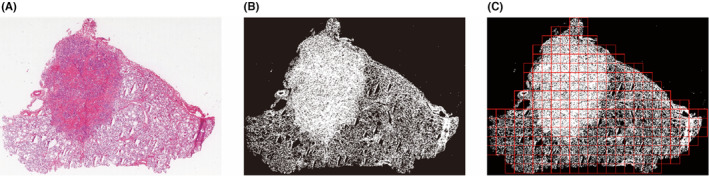
Examples of histopathology slide be processed before deep learning algorithm. A, original thyroid histopathology slide. B, processed to gray mask with threshold segmentation, the black area is background and will not be included by the algorithm. C, valid regions are cut into 3072 × 3072 pixels patches.

The background area in the slide typically makes up approximately 30%–60% of the entire slide, as shown in Figure 1A. When using computer vision algorithms for processing, the majority of the processing time is wasted processing the background area. These backgrounds are distinct from those of the useful regions. Almost no cells or organs were detected in the background. A computer vision threshold segmentation technique was used to extract areas containing cells and tissues from the image.^19^ As shown in Figure 1B, the dark areas are skipped directly without processing, which can speed up the algorithm and save 30%–60% of processing time. The cell tissue‐containing effective region was split into 3072 × 3072 size patches (Figure 1C).

To further improve the processing speed of the algorithm, 3072 × 3072 size patches were resized to 768 × 768 pixels, thereby reducing the number of pixels to be inferred by the algorithm by 75%.

### 2.2 | Benign and malignant subtype recognition algorithm

**FIGURE 2:**
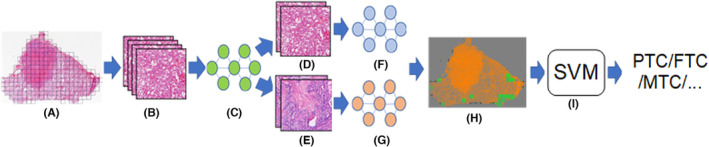
The process of AI algorithm inference thyroid histopathology slide. A, valid area in the slide has been cut into patches. B, 3072 × 3072 patches resized into 768 × 768 size. C, classifier for benign and malignant recognition. D, patches are judged as benign. E, patches are judged as malignant. F, classifier for benign subtype recognition. G, classifier for malignant subtype recognition. H, multi‐category lesions localization is accomplished with the results of classifiers, each color means a kind of subtype. I, slide‐level SVM classifier.

To judge benign and malignant subtypes in accordance with the outcomes of the malignant recognition algorithm and the benign subtype recognition algorithm, each processed patch in the pathological image was subjected to a deep learning algorithm for benign and malignant subtype recognition. The general movement of the algorithm is shown in Figure 2. All three of them, the recognition algorithms, are deep learning classifiers trained with annotations labeled by pathologists. All the model structures are Efficientnet_v2 (b0),^20^ which shows good performance based on the comprehensive performance evaluation of the experimental results. A three‐categorization image classification network that includes benign, malignant, and normal tissues makes up the benign and malignant recognition algorithm. The patches inferred as benign or malignant by the algorithm were further judged by the corresponding subtype recognition model.

A method for recognizing malignant subtypes, including PTC, FTC, MTC, P‐DTC, and ATC, was used to evaluate the patches that were determined to be malignant. The benign subtype identification method, which considers TA and TN, will also be used to evaluate benign patches. In addition, the localization of lesions and the slide‐level multi‐category identification will be accomplished using the recognition findings of all classes.

### 2.3 Slide‐level multi‐category recognition algorithm

The slide‐level multi‐category recognition algorithm is a non‐linear SVM whose input is the number sequence of each subtype under the same slide, including PTC, FTC, MTC, P‐DTC, ATC, TA, TN, and normal tissue. The essence of a non‐linear SVM is to map data samples to a high‐dimensional space and then maximize the interval; then, category gaps among each subtype are easily found.

### 2.4 Statistical analysis

There are some indexes used for experimental evaluation, which are defined as follows:

1. Precision: The fraction of relevant instances among all retrieved instances.
2. Recall: Sometimes referred to as sensitivity, it is the fraction of retrieved instances among all relevant instances.
3. F1‐score: Method for combining the precision and recall of the classifier, and it is defined as the harmonic mean of the classifier's precision and recall.
4. Sensitivity: The ability of a test to correctly identify patients with a disease.
5. Specificity: The ability of a test to correctly identify people without the disease.
6. PPV (Positive predictive value): The probability that a person with a positive test positive is a true positive.
7. Negative predictive value (NPV): The probability that a person who tests negative is true negative.
8. Accuracy: The proportion of correct predictions of a test.
9. Receiver operating characteristic (ROC) curves: Method to compare diagnostic tests. This is a plot of the true positive rate against the false positive rate.
10. Area under a curve (AUC): The area enclosed between the curve of a probability with non‐negative values and the axis of the quality being measured.

All the metrics above are calculated as follows:

(1) $$\text{Precision}=\frac{\mathrm{TP}}{\mathrm{TP}+\mathrm{FP}}$$

(2) $$\begin{array}{c} \text{Recall}=\text{Sensitivity}=\frac{\mathrm{TP}}{\mathrm{TP}+\mathrm{FN}} \end{array}$$

(3) $$\begin{array}{c} F1-\text{score}=\frac{2\times \text{recall}\times \text{precision}}{\text{recall}+\text{precision}} \end{array}$$

(4) $$\begin{array}{c} \text{Specificity}=\frac{\mathrm{TN}}{\mathrm{TN}+\mathrm{FP}} \end{array}$$

(5) $$\begin{array}{c} \mathrm{PPV}=\frac{\mathrm{TP}}{\mathrm{TP}+\mathrm{FP}} \end{array}$$

(6) $$\begin{array}{c} \mathrm{NPV}=\frac{\mathrm{TN}}{\mathrm{TN}+\mathrm{FN}} \end{array}$$

(7) $$\begin{array}{c} \text{Accuracy}=\frac{\mathrm{TP}+\mathrm{TN}}{\mathrm{TP}+\mathrm{TN}+\mathrm{FP}+\mathrm{FN}} \end{array}$$

## 3 RESULTS

### 3.1 Data collection

A total of 577 intraoperative frozen sections of the thyroid were collected and scanned into digital whole‐slide images (WSI). All the slides were scanned at 40× magnification (0.25 μm per pixel) by Digital Pathology Scanner (KF‐PRO‐400, KFBIO). Ethical approval for this study was granted by the Institutional Review Board of Sir Run Run Shaw Hospital, Zhejiang University School of Medicine (Approval No. 0415). Sensitive information such as the patient's name, medical record number, and ID number were removed from the digital slides.

The ground truth was based on formalin‐fixed paraffin‐embedded (FFPE) diagnosis rather than frozen section diagnosis, according to pathologists with expertise in thyroid disorders who annotated the 385 slides. We defined seven lesions (FTC, PTC, MTC, P‐DTC, ATC, TA, and TN), and pathologists drew polygons to circle the lesion area. Patients with Hürthle cell carcinoma, thyroid tumor of uncertain malignant potential (FT‐UMP), non‐invasive follicular thyroid neoplasm with papillary‐like nuclear features (NIFTP), and well‐differentiated neoplasms with uncertain malignant potential (WDT‐UMP) were excluded.

The annotated datasets were treated as training data, and the remaining 191 unannotated slides constituted the test dataset. After data processing, the training and test datasets were cut into 24153 and 14803 patches, respectively, and all the patches were in the annotation polygon area. The patient's clinical information and histopathologic results of the surgical procedures are listed in Table 1; *p* values <0.05 were considered statistically significant.

**TABLE 1:** Patient demographics according to training and test datasets.

	Training dataset (*n* = 376)	Test dataset (*n* = 191)	*p*value
Age, mean (SD), year	45.92 (14.63)	42.4 (14.28)	0.617
Malignant/benign	168/108	77/115	0.30
Sex, Female/Male	264/112	118/73	0.03
Compliance rate of routine diagnosis%	99.2	90.6	8.94e‐8
FTC (%)	69 (18.3)	48 (25.1)	0.02
PTC (%)	54 (14.3)	56 (29.3)	3.09e‐6
MTC (%)	34 (9.0)	5 (2.6)	3e‐3
P‐DTC (%)	7 (1.8)	7 (3.7)	0.18
ATC (%)	2 (0.5)	1 (0.5)	0.99
TA (%)	135 (35.9)	45 (23.5)	1.28e‐11
TN (%)	75 (19.9)	29 (15.2)	0.06

In these equations, TP is the true positive number determined by the classifier, TN is the true negative number, FP is the false positive number, and FN is the false negative number.

### 3.2 Patch‐level classification

We select several mainstream classification algorithm frameworks for experimental comparison, including Resnet50, Resnext50, SE Resnext50, InceptionV3, Efficientnet‐b0, and EfficientnetV2‐b0. In the training process of both patch‐level and slide‐level classifications, our deep learning model utilized pre‐trained weights from the ImageNet dataset to accelerate the convergence speed of the algorithm. Subsequently, they were trained using the training and validation datasets, and their performance was evaluated using the validation dataset. The learning rate of the algorithm is uniformly set to 1.0 × e −3, the optimizer is Adam, and the batch size is 32, with 60 training epochs. In addition, the training process also makes additional image augment operators, including random image rotation (50% trigger probability of rotation within ±30°), image flip (50% trigger probability of horizontal or vertical flip), hue saturation and value (HSV) augment (50% trigger probability within ±10%), and gaussian blur (kernel size = [5,5], *σ* = 0.1, and 50% trigger probability). All our algorithms were implemented using Python (version 3.6.10), PyTorch (version 1.8.0), scikit‐learn (version 0.18.3), opencv‐python (version 4.5.5.62), and openslide‐python (version 1.1.2). The categories of the training dataset were benign and malignant. The results are listed in Table 2. EfficientnetV2‐b0 has the highest F1‐score, and among model collections, its precision, recall, and sensitivity are higher. Besides, the AUC of ROC curves reflects that the classification performance of EfficientnetV2‐b0 is better than others. Therefore, efficientnetV2‐b0 was selected as the patch classifier in benign and malignant subtype classification.

**TABLE 2:** The performance of classification models in training.

Model	Precision%	Recall%	F1‐score%	Specificity%	AUC
Resnet50	82.3	61.2	70.2	83.3	74.3
Resnext50	86.4	57.8	69.2	86.8	70.1
SE‐Resnext50	89.2	54.4	67.6	87.9	70.3
InceptionV3	53.6	56.6	55.0	70.6	64.5
Efficientnet‐b0	75.6	68.5	71.8	82.8	77.2
EfficientnetV2‐b0	85.1	62.3	71.9	87.2	75.4

### 3.3 Slide‐level classification

A slide‐level classifier is a multi‐category SVM. In the data progress routine, patch‐level classifier inferences for all patches on the same slide are gathered, the number of each subtype's patches is encoded as a sequence, and the sequence is then normalized with all elements divided by the total number of patches into 0‐1.0. Then, as we defined seven types of lesions, the sequence is transformed into a 7×1 vector, which serves as the input and classified by slide‐level SVM, which offers a slide‐level classification result. During training, the SVM hyperparameters were iteratively updated using a Gaussian kernel function and a penalty index of 5.0. Hyperparameters generated by measuring the similarity between samples during training and in a space that portrays the similarity make the same samples better aligned and linearly separable.

To verify the performance and robustness, an unannotated test dataset including 45 TA, 48 FTC, 56 PTC, 5 MTC, 7 P‐DTC, 1 ATC, and 29 TN from the First Affiliated Hospital of Zhejiang University, the Second Affiliated Hospital of Zhejiang University, and Shaw Hospital Affiliated to Zhejiang University School of Medicine. The confusion matrix of benign and malignant nodules is listed in Table 3; no benign nodules were misclassified as malignant. The detailed result of the test dataset after SVM classification is listed in Table 4. The specificity is high in all types, and the AUC of PTC and MTC is higher than other types.

**TABLE 3:** Confusion matrix of testing dataset.

Confusion matrix		Ground truth						
		FTC (%)	PTC (%)	MTC (%)	P‐DTC (%)	ATC (%)	TN (%)	TA (%)
Predicted	FTC	11 (22.91)	0	0	1 (14.28)	0	0	0
	PTC	12 (25)	47 (83.92)	0	0	0	0	0
	MTC	0	1 (1.78)	5 (100)	1 (14.28)	0	0	0
	P‐DTC	0	1 (1.78)	0	5 (71.42)	1 (100)	0	0
	ATC	0	0	0	0	0	0	0
	TN	4 (8.33)	1 (1.78)	0	0	0	29 (100)	6 (13.33)
	TA	21 (43.75)	6 (10.71)	0	0	0	0	39 (86.67)

**TABLE 4:** Categorical classification result of SVM in test dataset. There is one case of ATC in test dataset, and is predicted as P‐DTC by SVM.

Category	Sensitivity %	Specificity %	Accuracy %	PPV %	NPV %	AUC
PTC	83.93	91.11	89.01	79.66	93.18	87.51
	(73.99–93.10)	(86.39–95.68)	(84.29–93.71)	(68.51–89.47)	(88.63–97.03)	(82.47–91.78)
FTC	22.92	99.30	80.10	91.67	79.33	61.10
	(10.42–34.14)	(97.43–100)	(73.82–85.34)	(70–100)	(73.14–85.00)	(53.65–67.24)
MTC	100	98.92	98.95	71.43	100	99.46
	(99.99–100.00)	(97.29–100.00)	(97.29–100.00)	(33.33–100.00)	(99.99–100.00)	(98.49–99.76)
P‐DTC	71.43	98.91	97.91	71.43	98.91	85.17
	(33.33–100.00)	(97.28–100.00)	(95.81–99.47)	(33.33–100.00)	(97.26–100.00)	(81.31–87.69)
ATC	0.00	100	99.48	0.00	99.48	50.00
	(0.00–0.00)	(100–100)	(99.37–99.61)	(0.00–0.00)	(99.37–99.61)	(50.00–50.00)
TA	86.67	81.51	82.72	59.09	95.20	96.60
	(75.55–95.99)	(74.99–87.50)	(76.96–87.95)	(45.94–72.13)	(90.97–98.47)	(92.41–97.13)
TN	100.0	93.21	94.24	72.50	100.00	84.08
	(99.99–100.00)	(89.17–96.81)	(91.09–97.38)	(58.53–86.04)	(99.99–100)	(82.37–87.14)
P‐DTC&ATC	75.00	99.45	98.43	85.71	98.91	87.22
	(39.99–100.00)	(98.32–100.00)	(96.33–100)	(49.99–100.00)	(97.25–100.00)	(80.26–90.13)
TA&TN	100.00	72.65	83.25	69.81	100	86.32
	(99.99–100)	(64.70–80.34)	(78.01–87.95)	(60.77–77.98)	(99.99–100)	(80.29–90.13)

*Note*: Therefore, the results of P‐DTC and ATC are summarized together. TN and TA are benign tumors; therefore, these two types of results are also summarized and presented. 95% confidence intervals are calculated by Bootstrap resampling method.

We processed histopathologic slides using a server with an Intel (R) Xeon (R) Gold 6326 CPU and Nvidia A100 80Gb GPU; one slide with 80000 × 80000 pixels was predicted to have an average of 237.6 seconds.

## 4 DISCUSSION

In this study, we developed an AI‐based system for accurately diagnosing thyroid nodules using various types of frozen sections. Our system can accurately localize tumors and provide pathologists with useful classification results. High AUC, sensitivity, and specificity scores were achieved by efficientnetV2‐b0 and SVM for the classification. This study has developed a trustworthy AI system for the accurate classification of thyroid nodules during IOPD, assisting pathologists and surgeons in improving diagnosis and designing surgical plans.

To the best of our knowledge, this study is the first to look into DL‐based pan‐thyroid disease classification in the frozen section. The choice of thyroidectomy surgery strategy is highly affected by the preoperative and intraoperative diagnoses. With the advancement of deep learning algorithms, many DL models have been developed for the preoperative diagnosis of thyroid nodules. Prior AI‐assisted thyroid tumor diagnostic studies primarily used images from medical radionics, including ultrasound, CT, and MRI images.^21^, ^22^, ^23^ These studies enhanced radiologists' competence, decreased overdiagnosis, and showed that AI was useful in assessing thyroid nodules. In addition, various AI technologies have been evaluated using thyroid cytological specimens. However, the DL model mainly focuses on benign and malignant classifications, and the malignant subtype classification is rarely involved.

This study aimed to develop an IOPD system to assist pathologists in diagnosing all types of thyroid diseases. Based on frozen section data, Yuan et al.^17^ developed an automated rule‐based method to distinguish between benign and malignant categories. In total, 95.3 percent of the benign nodules and 96.7 percent of the malignant nodules were correctly predicted using the algorithm. Zhu et al. used DL to diagnose thyroid nodules from frozen sections, however, that only worked for thyroid nodules that were classified as benign or malignant, and the FTC was defined as an unclear set with a 48.7% error rate.^24^ Among image recognition technologies, CNN stands out as the predominant algorithm, demonstrating superior performance when trained on extensive datasets.^25^ The CNN models utilized in this study—Resnet50, Resnext50, SE‐Resnext50, InceptionV3, Efficientnet‐b0, and EfficientnetV2‐b0—have been shown to be effective in identifying tumor pathology images in multiple studies. To select the optimal model, the effectiveness and learning potential of various models were assessed. In our system, FTC is partially classifiable, which achieved the AUC of 87.51 for PTC, 61.10 for FTC, and 99.46 for MTC. The specificity in FTC categorization is high at 99.30%, but the sensitivity is somewhat low, with 43.75% of FTC misclassified as TA. This is understandable given that FTC is usually diagnosed through a thorough screening for capsular or vascular invasion. TA categorization had a high sensitivity and specificity, and no TA were misclassified as malignant tumors. We additionally checked the WSI for Hashimoto's thyroiditis and discovered that it had not been misdiagnosed as PTC or any other cancer.

**FIGURE 3:**
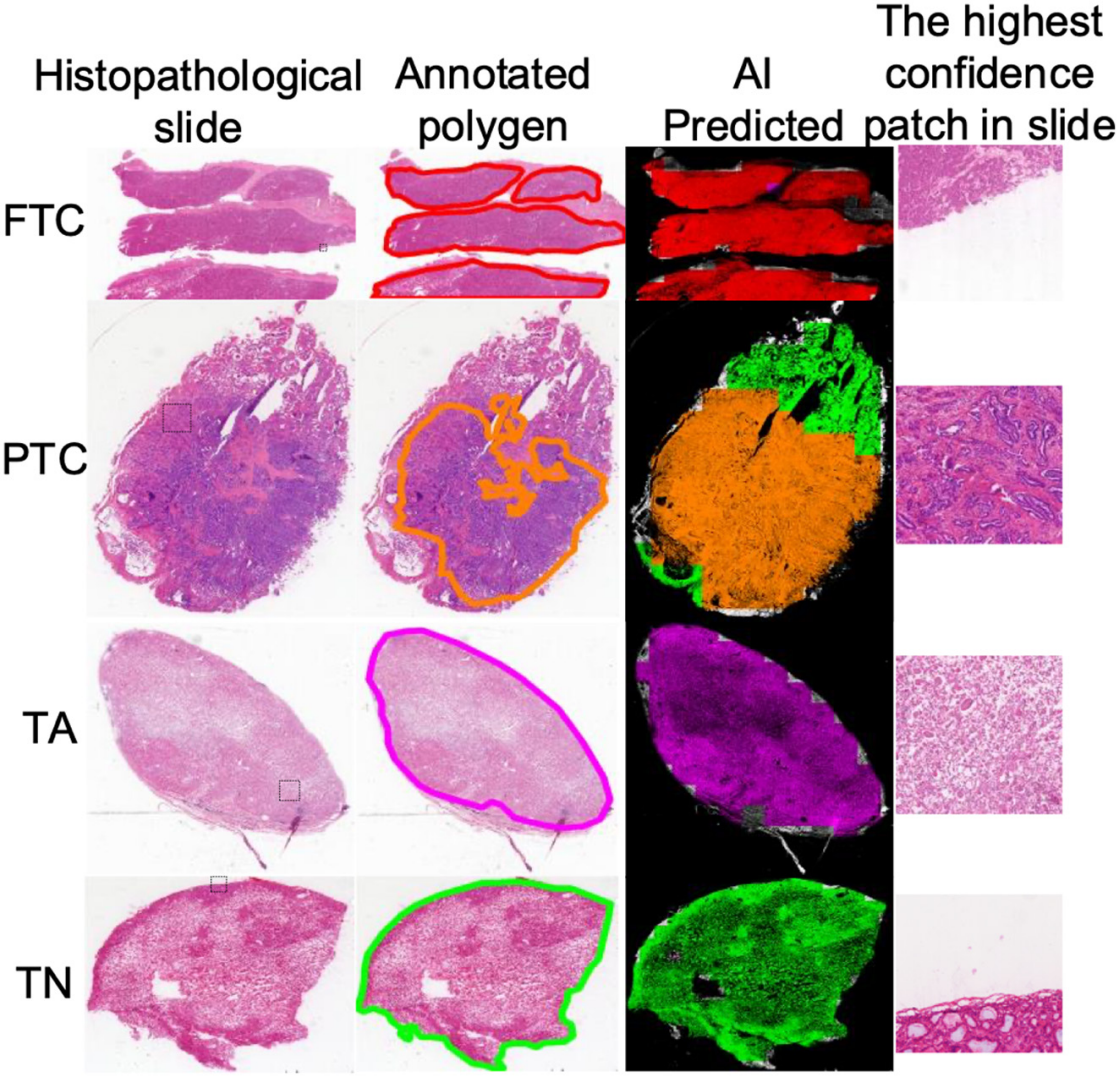
Visualization of patch‐level subtype prediction result in slides. In AI‐predicted result, the red region is FTC, the green region is TN, the pink region is TA, the orange region is PTC, the gray region is the area of normal tissue, and the black region is the uninterpreted background area. AI‐predicted results have a high overlap with the annotation. In addition, patch with the highest confidence index in slide has shown and the position within the slide have already been outlined with dashed box in the first column.

Subtype conclusions at the patch level can be mapped to various regions where the slides are predicted. As shown in Figure 3, we used different colors to represent the interpretation results at different positions on the slide. Figure 3 shows that we selected four samples for display: FTC, PTC, TA, and TN; each column includes the histopathological tissue slide, polygons marked on the tissue slide, visualization of the AI prediction results, and patch with the highest AI confidence in the tissue slide, which has already been outlined with a dashed box in the original slide column. The outcomes of the AI prediction were aligned with the annotated region. Visualization approaches can be used to enhance the confidence of the model and augment diagnostics, for example, by pinpointing the focus of pathologists on highly predictive areas (tiles) of a WSI or providing a matrix of the most predictive tiles.

**FIGURE 4:**
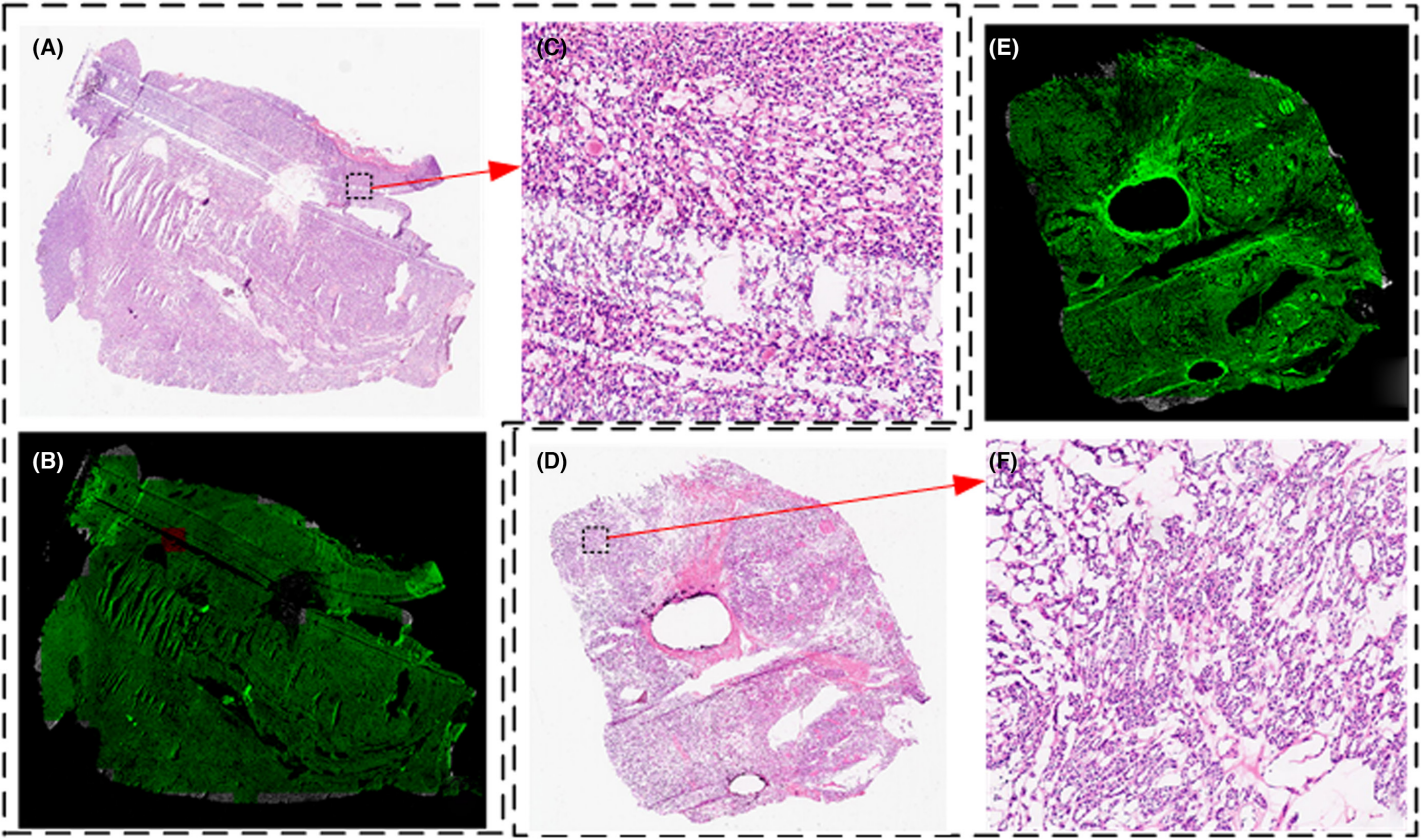
Visualization for analyzing misclassification results. A, FTC slide. B, AI results for A. C, 10× local magnification for A. D, TA slides. E, AI results for D. F, 10× local magnification for D.

In the test dataset, 32 cases of slides were misclassified as benign or malignant classifier. In patch‐level classification, firstly, all patches are classified as benign or malignant. Consequently, patch‐level subtypes are classified, and SVMs draw slide‐level conclusions based on the sequence of the number of patches under each subtype. If a slide has been classified incorrectly in the classification of benign and malignant at the patch‐level, it would not lead to a correct result in subtype classification, which will continue to affect the classification of slides by SVM. According to statistics, the average confidence of SVM output in the test dataset is 0.68, whereas the mean confidence of 32 cases (0.53) is lower than the mean confidence value. Besides, when we examined the test dataset, we observed that these tissue samples from FTC are not collected from the tissue surrounding the capsule, making it difficult to discern whether the capsule is invaded. The uncertainty rate of routine frozen section diagnosis is 90.6% in the test dataset; therefore, malignant conclusions cannot be drawn only from histopathology. On benign and malignant categories, the proposed method achieves sensitivity at 72.65% (64.70%–80.34%), specificity at 100.0% (99.99%–100.0%), and AUC at 86.32% (80.29%–90.13%) in the test dataset. As shown in Figure 4, 4(A) is the FTC slide that is diagnosed as TA by AI, and the capsule is unsampled, and 4(D) is the TA slide that has been diagnosed as TA. Comparing the local magnification shown in 4(C) and 4(F), the details are almost identical.

The pathological diagnosis of a frozen section is frequently offered for a little length of time, in contrast to the pathological diagnosis of a paraffin slice. One of the most important requirements for real‐time use of the DL model, which must be compatible with the accessibility of peripheral computer processing technologies, is its practical implementation. In the proposed method, all slides were preprocessed to recognize background areas that did not include any tissue; these were not predicted by classifiers and SVM. We observed that a slide could be predicted with a subtype of approximately 240 s. Approximately 120–160 s can be saved using a preprocessed slide, and background removal in the proposed method can improve the processing efficiency by approximately 40%. Our algorithm operates throughout the process in debug mode, and once it is packaged into software, it will accelerate even more. There is no denying that quantitative AI is part of the future of pathology. Nonetheless, the substantial expenses linked to scanning devices and data storage may presently pose a prohibitive barrier for impoverished, developing countries.

Still, our study has certain limitations. First, nodules with uncertain malignant potential such as, FT‐UMP, NIFTP, and WDT‐UMP, were excluded from the cohort. Second, the WSIs of frozen sections were scanned using the same scanner, and no particular techniques were utilized to cope with the various image types generated by different scanners. Third, this was a retrospective study; further prospective studies are needed to validate the accuracy and reliability of this AI model and evaluate its clinical utility in the real world.

In this study, we developed a patch‐based method that uses deep learning to automatically identify thyroid nodules in intraoperative frozen sections. We developed a two‐step model to combine all the patch predictions for thyroid nodule diagnosis. The first step was benign and malignant patch classification, and the second step was further subtype classification. The proposed system achieved an AUC of 86.32% for benign and malignant thyroid nodules. In addition, a typical whole‐slide image could be diagnosed within 240 s. The clinical consequences of using our system for the analysis of thyroid nodules can lead to more accurate and efficient diagnoses. Reducing interobserver variability and enhancing diagnostic reliability, AI models can also offer pathologists second opinions that are standardized and consistent. Clinically, the pathologist may take the result of AI model into consideration before send the diagnosis report. In future work, we will focus on enhancing the FTC and TA classification performance and expanding the system to include the uncertain malignant potential nodules.

## AUTHOR CONTRIBUTIONS

**Yan Ma:** Conceptualization (equal); data curation (equal); project administration (equal); resources (equal); writing – original draft (equal); writing – review and editing (equal). **Xiuming Zhang:** Data curation (equal); resources (equal); writing – review and editing (equal). **Zhongliang Yi:** Investigation (equal); resources (equal); supervision (equal); writing – review and editing (equal). **Liya Ding:** Data curation (equal); investigation (equal); resources (equal); writing – original draft (equal); writing – review and editing (equal). **Bojun Cai:** Software (equal); validation (equal); visualization (equal); writing – original draft (equal). **Zhinong Jiang:** Resources (equal); supervision (equal). **Wangwang Liu:** Formal analysis (equal); writing – review and editing (equal). **Hong Zou:** Conceptualization (equal); resources (equal); validation (equal). **Xiaomei Wang:** Resources (equal). **Guoxiang Fu:** Data curation (equal); project administration (equal); resources (equal); supervision (equal).

## CONFLICT OF INTEREST STATEMENT

The authors do not report any conflict of interest relevant to the present study. Participating centers contributed supplemental data under Institutional Review Board approval or waiver of consent where applicable.

## ETHICAL APPROVAL STATEMENT

Institutional review board of Sir Run Run Shaw Hospital, Zhejiang University School of Medicine approved this study on November 16, 2022 (Approval No. 0415).

## Data Availability

The data used in this study is unavailable due to privacy or restrictions and is the property of the Institute of Clinical Science, Department of Pathology, and Sir Run Run Shaw Hospital, therefore, cannot be shared.
